# CRISPR/Cas: a potential gene-editing tool in the nervous system

**DOI:** 10.1186/s13619-020-00044-6

**Published:** 2020-08-06

**Authors:** Yanxia Gao, Kexin Gao, Hui Yang

**Affiliations:** Institute of Neuroscience, State Key Laboratory of Neuroscience, Key Laboratory of Primate Neurobiology, CAS Center for Excellence in Brain Science and Intelligence Technology, Shanghai Research Center for Brain Science and Brain-Inspired Intelligence, Shanghai Institutes for Biological Sciences, Chinese Academy of Sciences, Shanghai, 200031 China

## Abstract

The rapidly developmental RNA-guided CRISPR/Cas system is a powerful tool for RNA and DNA editing in a variety of cells from different species and makes a great contribution to gene function research, disease model generation and gene therapy development in the past few years. The ease of use, low cost and high efficiency of CRISPR/Cas make it commonly used in various conditions. In this review, we introduce the CRISPR/Cas system and its diverse applications in nervous system briefly, which provides a better understanding for its potential application values.

## Background

The CRISPR/Cas system is gaining more and more popularity in gene editing and therapy since first discovered in 1987. Up to now, on one hand, different types of the CRISPR/Cas system were discovered to improve its size, editing efficiency and PAM limitations; on the other hand, by fusing different factors to the mutant Cas protein which inactivates its nuclease activity but retains its ability to bind a specific DNA target site by a guide RNA, different types of engineered CRISPR/Cas9 tools were developed to perform modification of a specific gene, like DNA methylation or demethylation, histone acetylation or deacetylation and so on. Here, we briefly introduce these tools and their applications in the nervous system.

## Main Text

### The CRISPR/Cas system

The Clustered regularly interspaced short palindromic repeats (CRISPR) and CRISPR-associated system (Cas) was first discovered in 1987 as a type of RNA-mediated adaptively immunity to defend foreign nucleotides in bacteria and archaea (Ishino et al. 1987; Wiedenheft et al. 2012) and later Charpentier, et al. revealed its potential of RNA-programmable genome editing in 2012 because of its ability of making site-specific DNA double-stranded breaks (DSBs) in vitro (Jinek et al. 2012). In the following year, researchers successfully used the CRISPR/Cas system to edit genome in mammalian cells, which indicated its strong application value in genome editing (Cho et al. 2013; Cong et al. 2013; Mali et al. 2013). Since then, the CRISPR/Cas system becomes more and more widely used in genomic editing because of its high efficiency, easy operation, low cost and diverse applications. Usually, CRISPR/Cas systems are divided into 2 classes, including 6 types (Makarova et al. 2015; Shmakov et al. 2017; Koonin et al. 2017; Pickar-Oliver and Gersbach 2019a), some of which are widely used as editing tools.

The class 2 type II CRISPR/Cas9 is one of the most popular editing tools, and simply composed of the CRISPR RNA (crRNA), the trans-activating crRNA (tracrRNA) and the Cas9 protein. In the engineered CRISPR/Cas system, it is usually simplified by transforming tracrRNA and crRNA into a chimeric single guide RNA (sgRNA) (Jinek et al. 2012), which can also direct the Cas9 to the target DNA sequence and cleave the DNA by recognizing the protospacer adjacent motif (PAM) based on Watson-Crick base pairing rules to edit genomic DNA through non-homologous end joining (NHEJ) or homology-directed repair (HDR) (Jinek et al. 2012; Cong et al. 2013; Mojica et al. 2009; Marraffini and Sontheimer 2010; Gasiunas et al. 2012; Doudna and Charpentier 2014). The *Streptococcus pyogenes* Cas9 (SpCas9, 1368 amino acids) is first used for genome editing by recognizing a simple 5′-NGG (N represents A, T, C or G) PAM (Jinek et al. 2012), however, its recognition limits the availability of SpCas9 targeting specific sites in the genome editing. To improve its availability, more Cas9 proteins from other species are found and engineered with various PAMs. For instance, *Staphylococcus aureus* Cas9 (SaCas9, 1053 amino acids), *Neisseria meningitidis* Cas9 (NmCas9, 1082 amino acids), S*treptococcus thermophilus* Cas9 (StCas9, 1121 amino acids) and *Campylobacter jejuni* Cas9 (CjCas9, 984 amino acids) respectively recognize the PAM of 5′-NNGRRT (R represents A or G), 5′-NNNNGATT, 5′-NNAGAAW(W represents A or T), and 5 -NNNVRYM (V represents A, C or G; Y represents C or T) (Cong et al. 2013; Esvelt et al. 2013; Zhang et al. 2013; Hou et al. 2013; Ran et al. 2015; Friedland et al. 2015; Yamada et al. 2017). The identification and improvement of Cas9 proteins that recognize different PAMs provides us more target sites for genome editing. In addition, RCas9 (O'Connell et al. 2014), SaCas9 (Strutt et al. 2018), CjCas9 (Dugar et al. 2018) and *Francisella novicida* Cas9 (FnCas9) (Sampson et al. 2013; Price et al. 2015) can also edit RNA at the same time.

In contrast with the CRISPR/Cas9 system, the Cas12a (known as Cpf1), which belongs to the Class 2 type V CRISPR-Cas system, is guided by the crRNA to the target site without the tracrRNA, and cleaves DNA in a staggered way by recognizing a T-rich PAM of 5′-TTN (Jinek et al. 2012; Garneau et al. 2010; Deltcheva et al. 2011; Chylinski et al. 2013; Zetsche et al. 2015). There are two Cas12a orthologues, *Acidaminococcus sp*. Cas12a (AsCas12a, 1307 amino acids) and *Lachnospiraceae bacterium* Cas12a (LbCas12a, 1228 amino acids), which have efficiency activity of genome editing in mammalian cells. The Cas12a can process its own crRNAs that makes it easier to target multiple sites (Zetsche et al. 2017), and has less off-target effects, compared to Cas9 (Kim et al. 2016). In conclusion, the CRISPR/Cas12a offers a choice of precise genomic modifications in the different applicational condition.

The recently discovered Cas13a is an RNA-targeting nuclease and belongs to class 2 type VI CRISPR/Cas13 system. The Cas13a contains two higher conservative eukaryotes and prokaryotes nucleotide-binding (HEPN) domains, which is guided by a crRNA to the target single-stranded RNA (ssRNA) and recognized by a H (H represents A, U or C) protospacer flanking sequence (PFS) in the 3′ end of the target sequence (Abudayyeh et al. 2016). Moreover, the Cas13b targets RNA to accomplish RNA cleavage by recognizing the double-sided PFS (Smargon et al. 2017). The Cas13d provides a higher efficiency of RNA targeting in various cells and organisms at the optimal cleavage temperature 24 ~ 41 °C (Konermann et al. 2018). In addition, the CasRx (Cas13d-NLS from *Ruminococcus flavefaciens* strain XPD3002) with a smaller size can be easily packaged into adeno-associated virus (AAV) and delivered to cells and organisms (Konermann et al. 2018), which expands the genome editing toolbox beyond DNA to RNA and plays a critical role in nucleic acid engineering, transcriptome-related study and therapy development.

### Mechanisms and applications of the CRISPR/Cas system in the nervous system

In the engineered CRISPR/Cas system, the Cas protein combining with sgRNA can target the specific gene loci and cut the DNA double-strand (DSB). Afterwards these DSBs are predominantly repaired by the error-prone non-homologous end joining (NHEJ) in eukaryotes (Wyman and Kanaar 2006; Pickar-Oliver and Gersbach 2019b), which bring about insertion or deletion (indels) in the target loci, in turn, result in gene inactivation. In addition, these DSBs are also repaired by homologous-directed recombination (HDR) and microhomology-mediated end joining (MMEJ) (Wyman and Kanaar 2006; Pickar-Oliver and Gersbach 2019b). If homologous donor sequence including our interesting sequence like gene markers tags or fluorescence protein are given during repair process, these markers possibly insert to sgRNA-targeted sites, which achieved site-specific insertion (Pickar-Oliver and Gersbach 2019b). As a result, as a new gene-editing tool, the CRISPR/Cas system has been being focused by more and more researchers including neuroscientists, since biological characterizations of the Cas protein were first found enabled to edit genes in 2012 (Jinek et al. 2012; Gasiunas et al. 2012).

The nervous system, as the most complex system in animals and humans, still has many mysteries in biology (Salles et al. 2019). For example, how does the nervous system especially the brain develop at embryo and which genes play essential roles in this process (Salles et al. 2019)? In addition, it is known that this system uses electrical and chemical means to help all parts of the body communicate with each other and performs many functions like sleep and wakefulness, mood, learning and memory, cognition and so on (Salles et al. 2019); however, many questions still need to be further explored, such as how neurons and genes in the brain achieve these functions. Besides, neuron typing also is a hard question because the number of neurons is very large, about 100 billion neurons in the humans brain (Herculano-Houzel 2009). When the CRISPR/Cas system is identified as an efficient gene-editing tools, researchers can hardly wait to explore its application in the nervous system and try their best to uncover these mysterious masks. In the following, we mainly review uses of the CRISPR/Cas system in the nervous system.

#### Knock-out and knock-in

Conventional methods of gene knock-out (KO) and knock-in (KI) like homologous recombination and LoxP/Cre-mediated conditional insertion and deletion are money and time consuming as they need to generate first chimeras and later KO or KI individuals (Doyle et al. 2012). Later, RNA interference enriches methods and saves time of gene knockout, although it still has some limitations, such as it cannot totally eliminate effects of a gene but knockdown gene expression level (Hannon 2002). However, the Cas-mediated tool shows competitive advantages in gene KO and KI due to its efficiency, complete deletion and time saving (Heidenreich and Zhang 2016).

Gene knock-out mediated by CRISPR/Cas in the nervous system are being reported. For example, CRISPR/sgRNA-mediated knockout successfully was achieved in induced pluripotent stem cells (iPSC)-derived neurons (Liu et al. 2016; Ortiz-Virumbrales et al. 2017), in brain slice neurons in vitro (Incontro et al. 2014) and in neurons in vivo (Shen et al. 2014; Swiech et al. 2015; Kalebic et al. 2016; Heman-Ackah et al. 2016; Park et al. 2019). Additionally, without limitations in the number of the gene loci, knockout of multiple genes, multi-copy genes and noncoding RNAs are achieved more easily than before. Typically, Amin, et al. identified functions of the multi-copy microRNA miR-218 in motor neurons by complete deletion with CRISPR/Cas (Amin et al. 2015). Moreover, it get easier access to knockout model for function identity of the gene in the nervous system, for example, Cdk5 roles in cortex folding and Mettl3 in neuronal differentiation were uncovered by its knockout with the help of CRISPR/Cas (Batista et al. 2014; Shinmyo et al. 2017). Moreover, CRISPR/Cas-mediated gene knockout dramatically saves time and money in construction of primate knockout model so that Prrt2-knockout and Bmal1-knockout monkeys were soon born in the Institute of Neuroscience (ION) from Chinese Academy of Sciences (Zuo et al. 2017; Qiu et al. 2019).

Specific insertion is becoming more convenient under the help of CRISPR/Cas. In the next year that CRISPR/Cas were used to edit genes, mice model carrying a fluorescent marker in the endogenous OCT4, NANOG, and SOX2 genes was one-step generated by injecting Cas9 mRNA, different sgRNAs and donor DNA vectors into zygotes (Yang et al. 2013), and mCherry knock-in monkey also was constructed (Yao et al. 2018). Another, Huntingtin knock-in pig model were also generated by CRISPR/Cas, which enables us to mimic the feature of Huntingtin neurodegeneration which is unavailable in the mice model (Yan et al. 2018a).

CRISPR/Cas are providing new strategies for study and therapy of neurological diseases, especially genetic disorders like Huntington’s disease (HD) and spinal muscular atrophy (SMA). HD is characterized by early striatal atrophy, which result from HTT level decreases in the brain due to CAG repeat expansion in huntingtin (HTT) gene (Jimenez-Sanchez et al. 2017). Earlier studies generated genetical HD mice models to simulate HD-like phenotypes (Mangiarini et al. 1996; Schilling et al. 1999; Ehrnhoefer et al. 2009), but there is still no efficient treatment for HD. However, researchers recently did not only generate HD pig model (Yan et al. 2018a), but also eliminated mutant HTT protein and relieved neuropathology by CRISPR/Cas-mediated inactivation of mutant HTT gene in vitro model (Shin et al. 2016; Kolli et al. 2017) and in mice model (Monteys et al. 2017; Yang et al. 2017), which provides promise for its cure. Moreover, SMA, along with general weakness and atrophy of spinal cord motor neurons and skeletal muscles, is also a severe autosomal recessive disease caused mainly by nucleotide mutations of the survival motor neuron 1 (SMN1) gene (Bergin et al. 1997; Schrank et al. 1997; Hamilton and Gillingwater 2013). However, its symptoms can be alleviated when its duplicate gene SMN2 is edited to increase SMN protein level by delivering CRISPR/Cas-sgRNA to SMA mice zygotes (Li et al. 2019), which give some cues for SMA treatment by CRISPR technology. Besides, researchers are exploring new strategies for other neurological disease like Parkinson’s disease by using the CRISPR system (Ortiz-Virumbrales et al. 2017; Park et al. 2019).

#### Base editing

Cytidine deaminase and adenine deaminase can convert cytosine into thymine (C to T) and adenine into guanine (A to G) separately. When they are fused to mutant Cas protein which can bind DNA but not cleave DNA double-strand, they can change base in the activity window of Cas protein, at the upstream of the protospacer adjacent motif (PAM) (Komor et al. 2016; Gaudelli et al. 2017; Molla and Yang 2019). Base editing is based on this principle. Considering PAM limitation to editing sites, besides discovering different Cas proteins in nature (Ma et al. 2019), numerous engineered Cas versions are also developed to extend scope of base editing, for example, the PAM of different SpCas9 variants SpCas9, VQR- SpCas9, VRER-SpCas9 and EQR-SpCas9 are separately 5′-NGG, 5′-NGAN or 5′- NGNG, 5′-NGCG and 5′-NGAG (Pickar-Oliver and Gersbach 2019b; Molla and Yang 2019; Kleinstiver et al. 2015). In addition, base editing activity also depends on deaminase which has different activities on a genomic site (Cheng et al. 2019). As a result, proper composition of Cas protein and deaminase is important for accurate and efficient base editing of different genes.

**Fig. 1:**
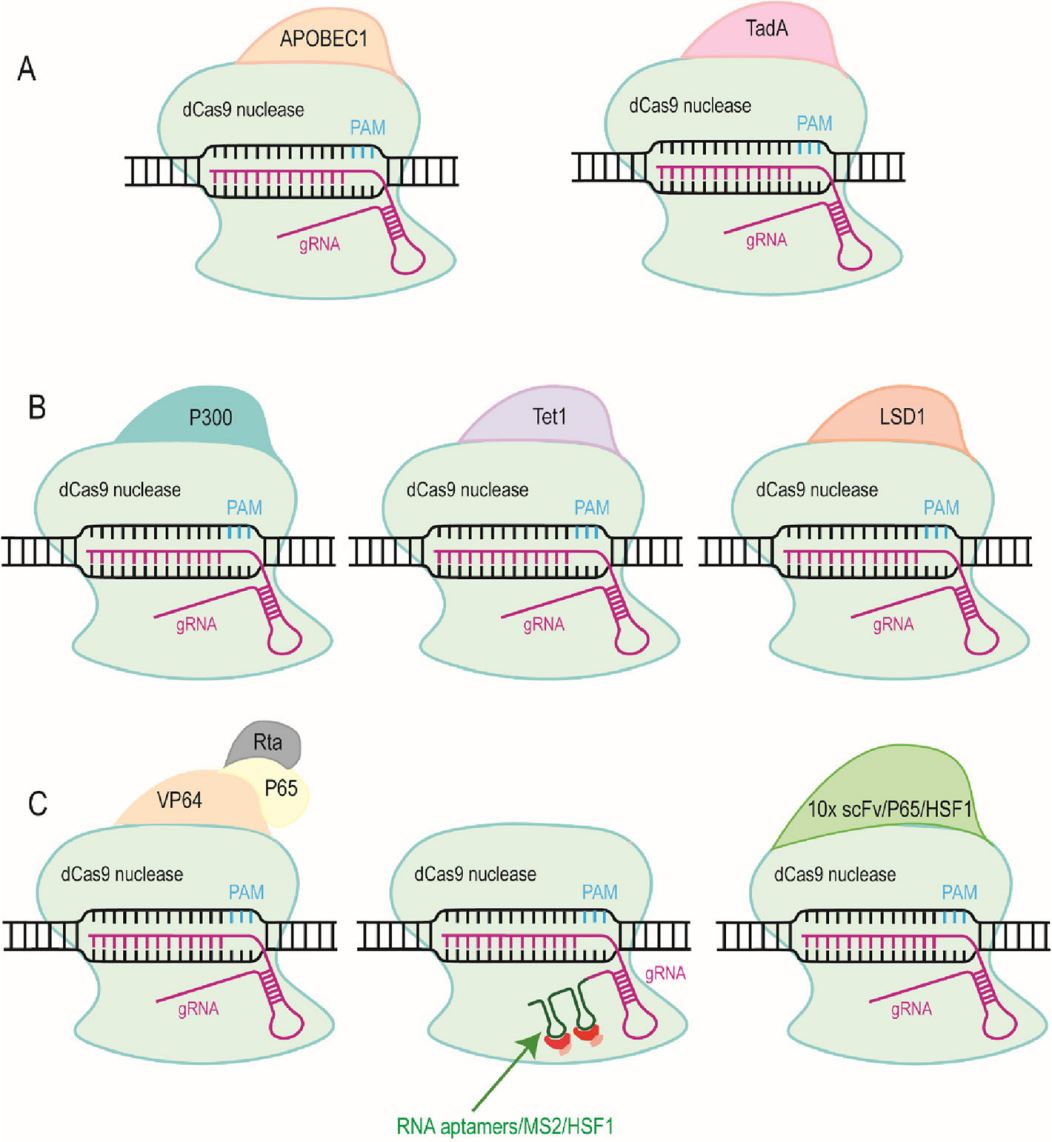
Schematic diagrams of the engineered Cas protein. **a** Base editors mediated by dead Cas9 (dCas9) with the separate fusion of the rat cytidine deaminase APOBEC1 and adenine deaminase TadA. **b** Specific epigenomic modification tools generated by dCas9 respectively fused with histone acetyltransferase P300, ten-eleven translocation methylcytosine dioxygenase 1 (Tet1) and lysine-specific histone demethylase 1 (LSD1). **c** Different CRISPR activation (CRISPRa) tools. Fusing three transcriptional activators VP64, p65 and Rta to the dCas protein at the same time can successfully activate multigene expression (left). Fusing two RNA hairpin aptamers which bind to dimers of the bacteriophage MS2 coat proteins and additional activators such as p65 and the human heat shock factor 1 (HSF1) to sgRNA can recruit more activation molecules (middle). Fusing 10 repeats of scFv (an activator module single-chain variable fragment) /p65/HSF1 to the dCas9 protein can more efficiently active multigene expression (right)

Noticeably, David Liu, et al. got up to 70% editing efficiency by separately fusing improved cytidine deaminase enzyme APOBEC1 and adenine deaminase TadA to mutant SpCas9 (also called dead Cas9, dCas9) (Fig. 1a), where they successfully converted an amino acid of APOE4, a gene related to Alzheimer’s disease in vitro (Komor et al. 2016; Gaudelli et al. 2017). Given no DSBs and accurate editing, engineered Cas-mediated point mutation shows potential advantages in correcting genetic diseases including neurological diseases. In addition, base editing provides a new strategy for inactivation of gene function by converting a protein-coding sequence into a stop codon (Zhang et al. 2018a).

#### Epigenomic modification

Epigenomic modifications like DNA methylation/demethylation, histone acetylation/deacetylation and nucleosome remodeling/positioning play important roles in neuronal differentiation and diseases (Feng et al. 2007; Zhou et al. 2018a). However, it is difficult to uncover how these epigenomic modifications of the specific gene loci affect gene functions in organisms with the conventional gene KO tool like the LoxP/Cre system because enzymes for epigenetic modification cannot target specific loci of genes, promotors or enhancers. CRISPR/Cas9 has recently emerged as an unparalleled tool for the interrogation of epigenome at individual loci, which is fused to different factors to complete different modification (Vora et al. 2016).

The dCas9-p300 system fusing dCas9 to histone acetyltransferase P300 developed by Hilton, et al. efficiently actives specific gRNA-targeted promotors and enhancers (Fig. 1b) (Hilton et al. 2015). In addition, dCas9 fusing to the catalytic domain of ten-eleven translocation methylcytosine dioxygenase 1 (Tet1) (Fig. 1b) or DNA methyltransferase DNMT3A efficiently methylate specific loci in vivo and in vitro (Valton et al. 2012; Vojta et al. 2016; McDonald et al. 2016), while dCas9 fusing to lysine-specific histone demethylase 1 (LSD1) (Fig. 1b) repressed expression of genes Oct4 and Tbx1 by targeting their enhancers, in turn to affected the cellular state of embryonic stem cells (Kearns et al. 2015). In conclusion, these systems have many potential applications in exploring epigenomic editing and regulation in neuronal development without causing global alterations (Zentner and Henikoff 2015; Pulecio et al. 2017).

Another epigenomic modification, RNA editing, is also enriched because of the discovery of the RNA-targeting Cas system such as the Cas13 protein. The Cas13 family can cut RNA strand by its nuclease domain, following targeting the specific loci mediated by the sgRNA (Cox et al. 2017; Yan et al. 2018b; Zhang et al. 2018b). Because of its small size, researchers show much interests in mRNA editing by the modified Cas13d protein. Cheng, et al. fused the splicing factor Fox1 element to dCas13d in order to inhibit Exon 7 skipping of SMN2 (Jillette and Cheng 2018), which gives new insights in SMA cures.

#### Regulation of gene expression

The dCas9 protein is also further fused to different transcription activation or inactivation domains, which constructs different CRISPR activation (CRISPRa) and CRISPR interference (CRISPRi) systems to regulate gene expression (Chavez et al. 2016). The most used CRISPRa system fused the dCas9 protein to three transcriptional activators VP64, p65 and Rta at the same time, which is proved successful to activate multigene expression to induce neuronal differentiation of human iPSCs (Fig. 1c) (Chavez et al. 2015). Moreover, sgRNAs also are modified by adding two RNA hairpin aptamers. They bind to dimers of the bacteriophage MS2 coat proteins which are fused to additional activators such as p65 and the human heat shock factor 1 (HSF1) to recruit more activation molecules for each dCas9 molecule and higher efficiently amplify multigene expression (Fig. 1c) (La Russa and Qi 2015). Zhou, et al. successfully achieved transcriptional activation of multiple genes in the mammalian developmental brain and efficiently converted astrocytes into functional neurons in vivo by improved CRISPRa systems (Zhou et al. 2018b). In the contrary, fusing the Krüppel-associated box of the transcriptional repressor Kox1 (Krab) to dCas9 can effective repress gene expression in cells (Fig. 1c) (Gilbert et al. 2013; Gilbert et al. 2014). Zheng, et al. used the CRISPRi tool to conditionally repress synaptotagmin I (Syt1) and found that the dentate gyrus of the hippocampus has distinct regulatory roles in learning and affective processes in mice (Zheng et al. 2018).

#### Inducible regulation

Fusing promotors like doxycycline- or light-dependent promotors to dCas9 achieves inducible regulation of Cas9 expression, in turn to regulate turn-on and turn-off of the Cas-based editing system. Doxycycline-inducible dCas9-based system has been used in reversible disease modelling in iPSC-derived cardiomyocytes, while the light-inducible dCas9-based system induced neuronal differentiation successfully (Mandegar et al. 2016; Nihongaki et al. 2017; Shao et al. 2018). This suggests that the inducible system achieves more precise regulation in specific gene expression in cell fate, neuronal differentiation or nervous disease.

#### High-throughput screen

The modified Cas9 system can efficiently change expression levels of large-scale genes through targeting them by sgRNA libraries, which makes a breakthrough to annotate functional characterizations of genetic elements in neuronal differentiation, normal neurobiological processes and diseases. Liu, et al. identified transcription factors that efficiently promote neuronal fate of ESCs by high-throughput CRISPRa screening with serial pooled sgRNA libararies, while Tian, et al. revealed neuron-specific roles of genes for survival, transcriptomics states, and morphology by CRISPRi-based knockdown with a pooled sgRNA libarary (Liu et al. 2018; Tian et al. 2019).

#### Future directions

CRISPR/Cas-based gene editing has been widely used in the nervous system because of its high efficiency, easy operation, low cost and diverse application, which help us uncover mysterious masks of various neurons and neuronal diseases. However, some potential risks still cannot be ignored. Firstly, DNA cleavage with the Cas endonuclease facilitates small insertions or deletions of nucleotides in unwanted sites (Zhang et al. 2015) and the cytosine/adenine deaminase may target non-specific RNA (Grunewald et al. 2019; Zhou et al. 2019; Zuo et al. 2019). These off-target editing may confound experiment results if it existed in a CRISPR-based animal model and could permanently disrupt normal gene function and lead to unpredictable complications if it is located in a patient’s DNA during CRISPR-based treatment. Secondly, considering the size of the Cas gene, it is difficult to enter cells directly. At present, Cas-sgRNA is addressed into cells mainly by liposome, virus vectors, ribonucleoprotein and so on, which have some drawbacks like limitation in size of the cargo, delivery efficiency and safety (Lino et al. 2018). In the future, more and more focus will be put on exploring hard neurobiological problems, correcting genetic diseases and performing cell therapies in the nervous system with CRISPR/Cas-based methods by overcoming its risks.

## References

[CR1] Abudayyeh OO, Gootenberg JS, Konermann S, Joung J, Slaymaker IM, Cox DB, Shmakov S, Makarova KS, Semenova E, Minakhin L, Severinov K, Regev A, Lander ES, Koonin EV, Zhang F (2016). C2c2 is a single-component programmable RNA-guided RNA-targeting CRISPR effector. Science.

[CR2] Amin ND, Bai G, Klug JR, Bonanomi D, Pankratz MT, Gifford WD, Hinckley CA, Sternfeld MJ, Driscoll SP, Dominguez B, Lee KF, Jin X, Pfaff SL (2015). Loss of motoneuron-specific microRNA-218 causes systemic neuromuscular failure. Science.

[CR3] Batista PJ, Molinie B, Wang J, Qu K, Zhang J, Li L, Bouley DM, Lujan E, Haddad B, Daneshvar K, Carter AC, Flynn RA, Zhou C, Lim KS, Dedon P, Wernig M, Mullen AC, Xing Y, Giallourakis CC, Chang HY (2014). m (6) A RNA modification controls cell fate transition in mammalian embryonic stem cells. Cell Stem Cell.

[CR4] Bergin A, Kim G, Price DL, Sisodia SS, Lee MK, Rabin BA (1997). Identification and characterization of a mouse homologue of the spinal muscular atrophy-determining gene, survival motor neuron. Gene.

[CR5] Chavez A, Scheiman J, Vora S, Pruitt BW, Tuttle M, PRI E, Lin S, Kiani S, Guzman CD, Wiegand DJ, Ter-Ovanesyan D, Braff JL, Davidsohn N, Housden BE, Perrimon N, Weiss R, Aach J, Collins JJ, Church GM (2015). Highly efficient Cas9-mediated transcriptional programming. Nat Methods.

[CR6] Chavez A, Tuttle M, Pruitt BW, Ewen-Campen B, Chari R, Ter-Ovanesyan D, Haque SJ, Cecchi RJ, Kowal EJK, Buchthal J, Housden BE, Perrimon N, Collins JJ, Church G (2016). Comparison of Cas9 activators in multiple species. Nat Methods.

[CR7] Cheng TL, Li S, Yuan B, Wang XL, Zhou WH, Qiu ZL (2019). Expanding C-T base editing toolkit with diversified cytidine deaminases. Nat Commun.

[CR8] Cho SW, Kim S, Kim JM, Kim JS (2013). Targeted genome engineering in human cells with the Cas9 RNA-guided endonuclease. Nat Biotechnol.

[CR9] Chylinski K, Le Rhun A, Charpentier E (2013). The tracrRNA and Cas9 families of type II CRISPR-Cas immunity systems. RNA Biol.

[CR10] Cong L, Ran FA, Cox D, Lin S, Barretto R, Habib N, Hsu PD, Wu X, Jiang W, Marraffini LA, Zhang F (2013). Multiplex genome engineering using CRISPR/Cas systems. Science.

[CR11] Cox DBT, Gootenberg JS, Abudayyeh OO, Franklin B, Kellner MJ, Joung J, Zhang F (2017). RNA editing with CRISPR-Cas13. Science.

[CR12] Deltcheva E, Chylinski K, Sharma CM, Gonzales K, Chao Y, Pirzada ZA, Eckert MR, Vogel J, Charpentier E (2011). CRISPR RNA maturation by trans-encoded small RNA and host factor RNase III. Nature.

[CR13] Doudna JA, Charpentier E (2014). Genome editing. The new frontier of genome engineering with CRISPR-Cas9. Science.

[CR14] Doyle A, McGarry MP, Lee NA, Lee JJ (2012). The construction of transgenic and gene knockout/knockin mouse models of human disease. Transgenic Res.

[CR15] Dugar G, Leenay RT, Eisenbart SK, Bischler T, Aul BU, Beisel CL, Sharma CM (2018). CRISPR RNA-dependent binding and cleavage of endogenous RNAs by the campylobacter jejuni Cas9. Mol Cell.

[CR16] Ehrnhoefer DE, Butland SL, Pouladi MA, Hayden MR (2009). Mouse models of Huntington disease: variations on a theme. Dis Model Mech.

[CR17] Esvelt KM, Mali P, Braff JL, Moosburner M, Yaung SJ, Church GM (2013). Orthogonal Cas9 proteins for RNA-guided gene regulation and editing. Nat Methods.

[CR18] Feng J, Fouse S, Fan G (2007). Epigenetic regulation of neural gene expression and neuronal function. Pediatr Res.

[CR19] Friedland AE, Baral R, Singhal P, Loveluck K, Shen S, Sanchez M, Marco E, Gotta GM, Maeder ML, Kennedy EM, Kornepati AV, Sousa A, Collins MA, Jayaram H, Cullen BR, Bumcrot D (2015). Characterization of Staphylococcus aureus Cas9: a smaller Cas9 for all-in-one adeno-associated virus delivery and paired nickase applications. Genome Biol.

[CR20] Garneau JE, Dupuis ME, Villion M, Romero DA, Barrangou R, Boyaval P, Fremaux C, Horvath P, Magadan AH, Moineau S (2010). The CRISPR/Cas bacterial immune system cleaves bacteriophage and plasmid DNA. Nature.

[CR21] Gasiunas G, Barrangou R, Horvath P, Siksnys V (2012). Cas9-crRNA ribonucleoprotein complex mediates specific DNA cleavage for adaptive immunity in bacteria. Proc Natl Acad Sci U S A.

[CR22] Gaudelli NM, Komor AC, Rees HA, Packer MS, Badran AH, Bryson DI, Liu DR (2017). Programmable base editing of A.T to G.C in genomic DNA without DNA cleavage. Nature.

[CR23] Gilbert LA, Horlbeck MA, Adamson B, Villalta JE, Chen Y, Whitehead EH, Guimaraes C, Panning B, Ploegh HL, Bassik MC, Qi LS, Kampmann M, Weissman JS (2014). Genome-scale CRISPR-mediated control of gene repression and activation. Cell.

[CR24] Gilbert LA, Larson MH, Morsut L, Liu Z, Brar GA, Torres SE, Stern-Ginossar N, Brandman O, Whitehead EH, Doudna JA, Lim WA, Weissman JS, Qi LS (2013). CRISPR-mediated modular RNA-guided regulation of transcription in eukaryotes. Cell.

[CR25] Grunewald J, Zhou R, Garcia SP, Iyer S, Lareau CA, Aryee MJ, Joung JK (2019). Transcriptome-wide off-target RNA editing induced by CRISPR-guided DNA base editors. Nature.

[CR26] Hamilton G, Gillingwater TH (2013). Spinal muscular atrophy: going beyond the motor neuron. Trends Mol Med.

[CR27] Hannon GJ (2002). RNA interference. Nature.

[CR28] Heidenreich M, Zhang F (2016). Applications of CRISPR-Cas systems in neuroscience. Nat Rev Neurosci.

[CR29] Heman-Ackah SM, Bassett AR, Wood MJ (2016). Precision modulation of neurodegenerative disease-related gene expression in human iPSC-derived neurons. Sci Rep.

[CR30] Herculano-Houzel S (2009). The human brain in numbers: a linearly scaled-up primate brain. Front Hum Neurosci.

[CR31] Hilton IB, D'Ippolito AM, Vockley CM, Thakore PI, Crawford GE, Reddy TE, Gersbach CA (2015). Epigenome editing by a CRISPR-Cas9-based acetyltransferase activates genes from promoters and enhancers. Nat Biotechnol.

[CR32] Hou Z, Zhang Y, Propson NE, Howden SE, Chu LF, Sontheimer EJ, Thomson JA (2013). Efficient genome engineering in human pluripotent stem cells using Cas9 from Neisseria meningitidis. Proc Natl Acad Sci U S A.

[CR33] Incontro S, Asensio CS, Edwards RH, Nicoll RA (2014). Efficient, complete deletion of synaptic proteins using CRISPR. Neuron.

[CR34] Ishino Y, Shinagawa H, Makino K, Amemura M, Nakata A (1987). Nucleotide sequence of the iap gene, responsible for alkaline phosphatase isozyme conversion in Escherichia coli, and identification of the gene product. J Bacteriol.

[CR35] Jillette N, Cheng A. CRISPR artificial splicing factors. BioRxiv. 2018. 10.1101/431064. 10.1038/s41467-020-16806-4 PMC7293279 32532987

[CR36] Jimenez-Sanchez M, Licitra F, Underwood BR, Rubinsztein DC. Huntington's disease: mechanisms of pathogenesis and therapeutic strategies. Cold Spring Harbor Perspect Med. 2017;7(7). 10.1101/cshperspect.a024240. 10.1101/cshperspect.a024240 PMC5495055 27940602

[CR37] Jinek M, Chylinski K, Fonfara I, Hauer M, Doudna JA, Charpentier E (2012). A programmable dual-RNA-guided DNA endonuclease in adaptive bacterial immunity. Science.

[CR38] Kalebic N, Taverna E, Tavano S, Wong FK, Suchold D, Winkler S, Huttner WB, Sarov M (2016). CRISPR/Cas9-induced disruption of gene expression in mouse embryonic brain and single neural stem cells in vivo. EMBO Rep.

[CR39] Kearns NA, Pham H, Tabak B, Genga RM, Silverstein NJ, Garber M, Maehr R (2015). Functional annotation of native enhancers with a Cas9-histone demethylase fusion. Nat Methods.

[CR40] Kim D, Kim J, Hur JK, Been KW, Yoon SH, Kim JS (2016). Genome-wide analysis reveals specificities of Cpf1 endonucleases in human cells. Nat Biotechnol.

[CR41] Kleinstiver BP, Prew MS, Tsai SQ, Topkar VV, Nguyen NT, Zheng ZL, Gonzales APW, Li ZY, Peterson RT, Yeh JRJ, Aryee MJ, Joung JK (2015). Engineered CRISPR-Cas9 nucleases with altered PAM specificities. Nature.

[CR42] Kolli N, Lu M, Maiti P, Rossignol J, Dunbar GL. CRISPR-Cas9 mediated gene-silencing of the mutant Huntingtin gene in an in vitro model of Huntington's disease. Int J Mol Sci. 2017;18(4). 10.3390/ijms18040754. 10.3390/ijms18040754 PMC5412339 28368337

[CR43] Komor AC, Kim YB, Packer MS, Zuris JA, Liu DR (2016). Programmable editing of a target base in genomic DNA without double-stranded DNA cleavage. Nature.

[CR44] Konermann S, Lotfy P, Brideau NJ, Oki J, Shokhirev MN, Hsu PD (2018). Transcriptome engineering with RNA-targeting type VI-D CRISPR effectors. Cell.

[CR45] Koonin EV, Makarova KS, Zhang F (2017). Diversity, classification and evolution of CRISPR-Cas systems. Curr Opin Microbiol.

[CR46] La Russa MF, Qi LS (2015). The new state of the art: Cas9 for gene activation and repression. Mol Cell Biol.

[CR47] Li J-J, Lin X, Tang C (2019). Disruption of splicing-regulatory elements using CRISPR/Cas9 rescues spinal muscular atrophy in human iPSCs and mice. Natl Sci Rev.

[CR48] Lino CA, Harper JC, Carney JP, Timlin JA (2018). Delivering CRISPR: a review of the challenges and approaches. Drug delivery.

[CR49] Liu J, Gao C, Chen W, Ma W, Li X, Shi Y, Zhang H, Zhang L, Long Y, Xu H, Guo X, Deng S, Yan X, Yu D, Pan G, Chen Y, Lai L, Liao W, Li Z (2016). CRISPR/Cas9 facilitates investigation of neural circuit disease using human iPSCs: mechanism of epilepsy caused by an SCN1A loss-of-function mutation. Transl Psychiatry.

[CR50] Liu YX, Yu C, Daley TP, Wang FY, Cao WS, Bhate S, Lin XQ, Still C, Liu HL, Zhao DH, Wang HF, Xie XMS, Ding S, Wong WH, Wernig M, Qi LS (2018). CRISPR activation screens systematically identify factors that drive neuronal fate and reprogramming. Cell Stem Cell.

[CR51] Ma DC, Xu ZM, Zhang ZY, Chen X, Zeng XZ, Zhang YY, Deng TY, Ren MF, Sun Z, Jiang R, Xie Z (2019). Engineer chimeric Cas9 to expand PAM recognition based on evolutionary information. Nat Commun.

[CR52] Makarova KS, Wolf YI, Alkhnbashi OS, Costa F, Shah SA, Saunders SJ, Barrangou R, Brouns SJ, Charpentier E, Haft DH, Horvath P, Moineau S, Mojica FJ, Terns RM, Terns MP, White MF, Yakunin AF, Garrett RA, van der Oost J, Backofen R, Koonin EV (2015). An updated evolutionary classification of CRISPR-Cas systems. Nat Rev Microbiol.

[CR53] Mali P, Yang L, Esvelt KM, Aach J, Guell M, DiCarlo JE, Norville JE, Church GM (2013). RNA-guided human genome engineering via Cas9. Science.

[CR54] Mandegar MA, Huebsch N, Frolov EB, Shin E, Truong A, Olvera MP, Chan AH, Miyaoka Y, Holmes K, Spencer CI, Judge LM, Gordon DE, Eskildsen TV, Villalta JE, Horlbeck MA, Gilbert LA, Krogan NJ, Sheikh SP, Weissman JS, Qi LS, So PL, Conklin BR (2016). CRISPR interference efficiently induces specific and reversible gene silencing in human iPSCs. Cell Stem Cell.

[CR55] Mangiarini L, Sathasivam K, Seller M, Cozens B, Harper A, Hetherington C, Lawton M, Trottier Y, Lehrach H, Davies SW, Bates GP (1996). Exon 1 of the HD gene with an expanded CAG repeat is sufficient to cause a progressive neurological phenotype in transgenic mice. Cell.

[CR56] Marraffini LA, Sontheimer EJ (2010). CRISPR interference: RNA-directed adaptive immunity in bacteria and archaea. Nat Rev Genet.

[CR57] McDonald JI, Celik H, Rois LE, Fishberger G, Fowler T, Rees R, Kramer A, Martens A, Edwards JR, Challen GA (2016). Reprogrammable CRISPR/Cas9-based system for inducing site-specific DNA methylation. Biology open.

[CR58] Mojica FJ, Diez-Villasenor C, Garcia-Martinez J, Almendros C (2009). Short motif sequences determine the targets of the prokaryotic CRISPR defence system. Microbiology.

[CR59] Molla KA, Yang Y (2019). CRISPR/Cas-Mediated base editing: technical considerations and practical applications. Trends Biotechnol.

[CR60] Monteys AM, Ebanks SA, Keiser MS, Davidson BL (2017). CRISPR/Cas9 editing of the mutant Huntingtin allele in vitro and in vivo. Mol Ther.

[CR61] Nihongaki Y, Furuhata Y, Otabe T, Hasegawa S, Yoshimoto K, Sato M (2017). CRISPR-Cas9-based photoactivatable transcription systems to induce neuronal differentiation. Nat Methods.

[CR62] O'Connell MR, Oakes BL, Sternberg SH, East-Seletsky A, Kaplan M, Doudna JA (2014). Programmable RNA recognition and cleavage by CRISPR/Cas9. Nature.

[CR63] Ortiz-Virumbrales M, Moreno CL, Kruglikov I, Marazuela P, Sproul A, Jacob S, Zimmer M, Paull D, Zhang B, Schadt EE, Ehrlich ME, Tanzi RE, Arancio O, Noggle S, Gandy S (2017). CRISPR/Cas9-correctable mutation-related molecular and physiological phenotypes in iPSC-derived Alzheimer's PSEN2 (N141I) neurons. Acta Neuropathologica Communications.

[CR64] Park H, Oh J, Shim G, Cho B, Chang Y, Kim S, Baek S, Kim H, Shin J, Choi H, Yoo J, Kim J, Jun W, Lee M, Lengner CJ, Oh YK, Kim J (2019). In vivo neuronal gene editing via CRISPR-Cas9 amphiphilic nanocomplexes alleviates deficits in mouse models of Alzheimer's disease. Nat Neurosci.

[CR65] Pickar-Oliver A, Gersbach CA (2019). The next generation of CRISPR-Cas technologies and applications. Nat Rev Mol Cell Biol.

[CR66] Price AA, Sampson TR, Ratner HK, Grakoui A, Weiss DS (2015). Cas9-mediated targeting of viral RNA in eukaryotic cells. Proc Natl Acad Sci U S A.

[CR67] Pulecio J, Verma N, Mejia-Ramirez E, Huangfu D, Raya A (2017). CRISPR/Cas9-based engineering of the Epigenome. Cell Stem Cell.

[CR68] Qiu PY, Jiang J, Liu Z, Cai YJ, Huang T, Wang Y, Liu QM, Nie YH, Liu F, Cheng JM, Li Q, Tang YC, Poo MM, Sun Q, Chang HC (2019). BMAL1 knockout macaque monkeys display reduced sleep and psychiatric disorders. Natl Sci Rev.

[CR69] Ran FA, Cong L, Yan WX, Scott DA, Gootenberg JS, Kriz AJ, Zetsche B, Shalem O, Wu X, Makarova KS, Koonin EV, Sharp PA, Zhang F (2015). In vivo genome editing using Staphylococcus aureus Cas9. Nature.

[CR70] Salles A, Bjaalie JG, Evers K, Farisco M, Fothergill BT, Guerrero M, Maslen H, Muller J, Prescott T, Stahl BC, Walter H, Zilles K, Amunts K (2019). The human brain project: responsible brain research for the benefit of society. Neuron.

[CR71] Sampson TR, Saroj SD, Llewellyn AC, Tzeng YL, Weiss DS (2013). A CRISPR/Cas system mediates bacterial innate immune evasion and virulence. Nature.

[CR72] Schilling G, Becher MW, Sharp AH, Jinnah HA, Duan K, Kotzuk JA, Slunt HH, Ratovitski T, Cooper JK, Jenkins NA, Copeland NG, Price DL, Ross CA, Borchelt DR (1999). Intranuclear inclusions and neuritic aggregates in transgenic mice expressing a mutant N-terminal fragment of huntingtin. Hum Mol Genet.

[CR73] Schrank B, Gotz R, Gunnersen JM, Ure JM, Toyka KV, Smith AG, Sendtner M (1997). Inactivation of the survival motor neuron gene, a candidate gene for human spinal muscular atrophy, leads to massive cell death in early mouse embryos. Proc Natl Acad Sci U S A.

[CR74] Shao J, Wang M, Yu G, Zhu S, Yu Y, Heng BC, Wu J, Ye H (2018). Synthetic far-red light-mediated CRISPR-dCas9 device for inducing functional neuronal differentiation. Proc Natl Acad Sci U S A.

[CR75] Shen Z, Zhang X, Chai Y, Zhu Z, Yi P, Feng G, Li W, Ou G (2014). Conditional knockouts generated by engineered CRISPR-Cas9 endonuclease reveal the roles of coronin in C. elegans neural development. Dev Cell.

[CR76] Shin JW, Kim KH, Chao MJ, Atwal RS, Gillis T, MacDonald ME, Gusella JF, Lee JM (2016). Permanent inactivation of Huntington's disease mutation by personalized allele-specific CRISPR/Cas9. Hum Mol Genet.

[CR77] Shinmyo Y, Terashita Y, Dinh Duong TA, Horiike T, Kawasumi M, Hosomichi K, Tajima A, Kawasaki H (2017). Folding of the cerebral cortex requires Cdk5 in upper-layer neurons in Gyrencephalic mammals. Cell Rep.

[CR78] Shmakov S, Smargon A, Scott D, Cox D, Pyzocha N, Yan W, Abudayyeh OO, Gootenberg JS, Makarova KS, Wolf YI, Severinov K, Zhang F, Koonin EV (2017). Diversity and evolution of class 2 CRISPR-Cas systems. Nat Rev Microbiol.

[CR79] Smargon AA, Cox DBT, Pyzocha NK, Zheng K, Slaymaker IM, Gootenberg JS, Abudayyeh OA, Essletzbichler P, Shmakov S, Makarova KS, Koonin EV, Zhang F (2017). Cas13b is a type VI-B CRISPR-associated RNA-guided RNase differentially regulated by accessory proteins Csx27 and Csx28. Mol Cell.

[CR80] Strutt SC, Torrez RM, Kaya E, Negrete OA, Doudna JA (2018) RNA-dependent RNA targeting by CRISPR-Cas9. eLife 7. doi:10.7554/eLife.32724. 10.7554/eLife.32724 PMC5796797 29303478

[CR81] Swiech L, Heidenreich M, Banerjee A, Habib N, Li YQ, Trombetta J, Sur M, Zhang F (2015). In vivo interrogation of gene function in the mammalian brain using CRISPR-Cas9. Nat Biotechnol.

[CR82] Tian R, Gachechiladze MA, Ludwig CH, Laurie MT, Hong JY, Nathaniel D, Prabhu AV, Fernandopulle MS, Patel R, Abshari M, Ward ME, Kampmann M. CRISPR interference-based platform for multimodal genetic screens in human iPSC-derived neurons. Neuron. 2019. 10.1016/j.neuron.2019.07.014. 10.1016/j.neuron.2019.07.014 PMC6813890 31422865

[CR83] Valton J, Dupuy A, Daboussi F, Thomas S, Marechal A, Macmaster R, Melliand K, Juillerat A, Duchateau P. Overcoming transcription activator-like effector (TALE) DNA binding domain sensitivity to cytosine methylation. J Biol Chem. 2012;287(46):38427–32. 10.1074/jbc.C112.408864. 10.1074/jbc.C112.408864 PMC3493886 23019344

[CR84] Vojta A, Dobrinic P, Tadic V, Bockor L, Korac P, Julg B, Klasic M, Zoldos V (2016). Repurposing the CRISPR-Cas9 system for targeted DNA methylation. Nucleic Acids Res.

[CR85] Vora S, Tuttle M, Cheng J, Church G (2016). Next stop for the CRISPR revolution: RNA-guided epigenetic regulators. FEBS J.

[CR86] Wiedenheft B, Sternberg SH, Doudna JA (2012). RNA-guided genetic silencing systems in bacteria and archaea. Nature.

[CR87] Wyman C, Kanaar R (2006). DNA double-strand break repair: all's well that ends well. Annu Rev Genet.

[CR88] Yamada M, Watanabe Y, Gootenberg JS, Hirano H, Ran FA, Nakane T, Ishitani R, Zhang F, Nishimasu H, Nureki O (2017). Crystal structure of the minimal Cas9 from campylobacter jejuni reveals the molecular diversity in the CRISPR-Cas9 systems. Mol Cell.

[CR89] Yan S, Tu ZC, Liu ZM, Fan NN, Yang HM, Yang S, Yang WL, Zhao Y, Ouyang Z, Lai CD, Yang HQ, Li L, Liu QS, Shi H, Xu GQ, Zhao H, Wei HJ, Pei Z, Li SH, Lai LX, Li XJ (2018). A Huntingtin Knockin pig model recapitulates features of selective Neurodegeneration in Huntington's disease. Cell.

[CR90] Yan WX, Chong SR, Zhang HB, Makarova KS, Koonin EV, Cheng DR, Scott DA (2018). Cas13d is a compact RNA-targeting type VI CRISPR effector positively modulated by a WYL-domain-containing accessory protein. Mol Cell.

[CR91] Yang H, Wang H, Shivalila CS, Cheng AW, Shi L, Jaenisch R (2013). One-step generation of mice carrying reporter and conditional alleles by CRISPR/Cas-mediated genome engineering. Cell.

[CR92] Yang S, Chang RB, Yang HM, Zhao T, Hong Y, Kong HE, Sun XB, Qin ZH, Jin P, Li SH, Li XJ (2017). CRISPR/Cas9-mediated gene editing ameliorates neurotoxicity in mouse model of Huntington's disease. J Clin Invest.

[CR93] Yao X, Liu Z, Wang X, Wang Y, Nie YH, Lai L, Sun RL, Shi LY, Sun Q, Yang H (2018). Generation of knock-in cynomolgus monkey via CRISPR/Cas9 editing. Cell Res.

[CR94] Zentner GE, Henikoff S (2015). Epigenome editing made easy. Nat Biotechnol.

[CR95] Zetsche B, Gootenberg JS, Abudayyeh OO, Slaymaker IM, Makarova KS, Essletzbichler P, Volz SE, Joung J, van der Oost J, Regev A, Koonin EV, Zhang F (2015). Cpf1 is a single RNA-guided endonuclease of a class 2 CRISPR-Cas system. Cell.

[CR96] Zetsche B, Heidenreich M, Mohanraju P, Fedorova I, Kneppers J, DeGennaro EM, Winblad N, Choudhury SR, Abudayyeh OO, Gootenberg JS, Wu WY, Scott DA, Severinov K, van der Oost J, Zhang F (2017). Multiplex gene editing by CRISPR-Cpf1 using a single crRNA array. Nat Biotechnol.

[CR97] Zhang C, Konermann S, Brideau NJ, Lotfy P, Wu XB, Novick SJ, Strutzenberg T, Griffin PR, Hsu PD, Lyumkis D (2018). Structural basis for the RNA-guided Ribonuclease activity of CRISPR-Cas13d. Cell.

[CR98] Zhang H, Pan H, Zhou CY, Wei Y, Ying WQ, Li ST, Wang GQ, Li C, Ren YF, Li G, Ding X, Sun YD, Li GL, Song L, Li YX, Yang H, Liu ZY (2018). Simultaneous zygotic inactivation of multiple genes in mouse through CRISPR/Cas9-mediated base editing. Development.

[CR99] Zhang XH, Tee LY, Wang XG, Huang QS, Yang SH (2015). Off-target effects in CRISPR/Cas9-mediated genome engineering. Mol Ther Nucleic Acids.

[CR100] Zhang Y, Heidrich N, Ampattu BJ, Gunderson CW, Seifert HS, Schoen C, Vogel J, Sontheimer EJ (2013). Processing-independent CRISPR RNAs limit natural transformation in Neisseria meningitidis. Mol Cell.

[CR101] Zheng Y, Shen W, Zhang J, Yang B, Liu YN, Qi H, Yu X, Lu SY, Chen Y, Xu YZ, Li Y, Gage FH, Mi S, Yao J (2018). CRISPR interference-based specific and efficient gene inactivation in the brain. Nat Neurosci.

[CR102] Zhou C, Sun Y, Yan R, Liu Y, Zuo E, Gu C, Han L, Wei Y, Hu X, Zeng R, Li Y, Zhou H, Guo F, Yang H. Off-target RNA mutation induced by DNA base editing and its elimination by mutagenesis. Nature. 2019. 10.1038/s41586-019-1314-0. 10.1038/s41586-019-1314-0 31181567

[CR103] Zhou H, Wang B, Sun H, Xu X, Wang Y (2018). Epigenetic regulations in neural stem cells and neurological diseases. Stem Cells Int.

[CR104] Zhou HB, Liu JL, Zhou CY, Gao N, Rao ZP, Li H, Hu XD, Li CL, Yao X, Shen XW, Sun YD, Wei Y, Liu F, Ying WQ, Zhang JM, Tang C, Zhang X, Xu HT, Shi LY, Cheng LP, Huang PY, Yang H (2018). In vivo simultaneous transcriptional activation of multiple genes in the brain using CRISPR-dCas9-activator transgenic mice. Nat Neurosci.

[CR105] Zuo E, Sun Y, Wei W, Yuan T, Ying W, Sun H, Yuan L, Steinmetz LM, Li Y, Yang H (2019). Cytosine base editor generates substantial off-target single-nucleotide variants in mouse embryos. Science.

[CR106] Zuo EW, Cai YJ, Li K, Wei Y, Wang BA, Sun YD, Liu Z, Liu JW, Hu XD, Wei W, Huo XN, Shi LY, Tang C, Liang D, Wang Y, Nie YH, Zhang CC, Yao X, Wang X, Zhou CY, Ying WQ, Wang QF, Chen RC, Shen Q, Xu GL, Li JS, Sun Q, Xiong ZQ, Yang H (2017). One-step generation of complete gene knockout mice and monkeys by CRISPR/Cas9-mediated gene editing with multiple sgRNAs. Cell Res.

