# Correction to: CRISPR/Cas: a potential gene-editing tool in the nervous system

**DOI:** 10.1186/s13619-021-00095-3

**Published:** 2021-10-13

**Authors:** Yanxia Gao, Kexin Gao, Hui Yang

**Affiliations:** Institute of Neuroscience, State Key Laboratory of Neuroscience, Key Laboratory of Primate Neurobiology, CAS Center for Excellence in Brain Science and Intelligence Technology, Shanghai Research Center for Brain Science and Brain-Inspired Intelligence, Shanghai Institutes for Biological Sciences, Chinese Academy of Sciences, Shanghai, 200031 China

**Correction to: Cell Regeneration (2020) 9:12**

https://doi.org/10.1186/s13619-020–00,044-6

Following publication of the original article (Gao et al. 2020 [1]), it is reported that the “Background” section and the heading “Main Text” need to be added to the article.

The Background section has been provided below.

Background

The CRISPR/Cas system is gaining more and more popularity in gene editing and therapy since first discovered in 1987. Up to now, on one hand, different types of the CRISPR/Cas system were discovered to improve its size, editing efficiency and PAM limitations; on the other hand, by fusing different factors to the mutant Cas protein which inactivates its nuclease activity but retains its ability to bind a specific DNA target site by a guide RNA, different types of engineered CRISPR/Cas9 tools were developed to perform modification of a specific gene, like DNA methylation or demethylation, histone acetylation or deacetylation and so on. Here, we briefly introduce these tools and their applications in the nervous system.

The original article (Gao et al. 2020 [1]) has been updated.
